# The Differential Metabolomes in Cumulus and Mural Granulosa Cells from Human Preovulatory Follicles

**DOI:** 10.1007/s43032-021-00691-3

**Published:** 2021-08-10

**Authors:** Er-Meng Gao, Bongkoch Turathum, Ling Wang, Di Zhang, Yu-Bing Liu, Rong-Xin Tang, Ri-Cheng Chian

**Affiliations:** 1 Shanghai Clinical College, Anhui Medical University, Hefei, People’s Republic of China; 2 Department of Basic Medical Science, Faculty of Medicine Vajira Hospital, Navamindradhiraj University, Bangkok, Thailand; 3 Centre for Reproductive Medicine, Shanghai 10th People Hospital of Tongji University, Shanghai, People’s Republic of China

**Keywords:** Mural granulosa cells, Cumulus cells, Metabolomes, Human, Follicle

## Abstract

This study evaluated the differences in metabolites between cumulus cells (CCs) and mural granulosa cells (MGCs) from human preovulatory follicles to understand the mechanism of oocyte maturation involving CCs and MGCs. CCs and MGCs were collected from women who were undergoing in vitro fertilization (IVF)/intracytoplasmic sperm injection (ICSI) treatment. The differences in morphology were determined by immunofluorescence. The metabolomics of CCs and MGCs was measured by liquid chromatography coupled to tandem mass spectrometry (LC-MS/MS) followed by quantitative polymerase chain reaction (qPCR) and western blot analysis to further confirm the genes and proteins involved in oocyte maturation. CCs and MGCs were cultured for 48 h in vitro, and the medium was collected for detection of hormone levels. There were minor morphological differences between CCs and MGCs. LC-MS/MS analysis showed that there were differences in 101 metabolites between CCs and MGCs: 7 metabolites were upregulated in CCs, and 94 metabolites were upregulated in MGCs. The metabolites related to cholesterol transport and estradiol production were enriched in CCs, while metabolites related to antiapoptosis were enriched in MGCs. The expression of genes and proteins involved in cholesterol transport (ABCA1, LDLR, and SCARB1) and estradiol production (SULT2B1 and CYP19A1) was significantly higher in CCs, and the expression of genes and proteins involved in antiapoptosis (CRLS1, LPCAT3, and PLA2G4A) was significantly higher in MGCs. The level of estrogen in CCs was significantly higher than that in MGCs, while the progesterone level showed no significant differences. There are differences between the metabolomes of CCs and MGCs. These differences may be involved in the regulation of oocyte maturation.

## Introduction

Granulosa cells (GCs) are somatic cells surrounding oocytes that differentiate into cumulus cells (CCs) and mural granulosa cells (MGCs) during the antral follicular phase. CCs and oocytes directly contact one another to form a cumulus-oocyte complex (COCs), which mainly provides nutrition and energy to the oocytes, while MGCs are arranged on the wall of the follicle and mainly perform endocrine functions. Both CCs and MGCs play an important role in the growth and development of follicles [1, 2].

Oocyte quality is directly related to the microenvironment in follicles associated with CCs and MGCs. According to previous studies, the increased gene expression of amphiregulin (AREG), cyclooxygenase 2 (PTGS2), steroidogenic acute regulatory protein (STAR), stearoyl-coenzyme A desaturase 1 (SCD1), and SCD5 in CCs is associated with the nuclear maturation of oocytes. Among them, STAR, PTGS2, and AREG are related to the LH peak, while SCD1 and SCD5 are related to the lipid metabolism of oocytes [3]. In humans, the expression of versican (VCAN) and PTGS2 in CCs is significantly higher, which leads to pregnancy and live birth. It has been demonstrated that the efficacy of cumulus-assisted embryo transfer using autologous CCs yields a significant increase in implantation and pregnancy rates [4]. Fan et al. [5] found that the apoptosis rate of MGCs in women with hypoovarian function was significantly higher than that in women with normal ovarian reserve.

Metabolomics is the downstream complement of genomics, transcriptomics, and proteomics, providing a comprehensive assessment of the physiological state of biological systems. Metabolomics allows the identification and quantification of small molecules. Among many analytical techniques, LC-MS technology is widely used in targeted and nontargeted metabolites analysis, allows more polar compounds to be identified, and is the gold standard for analyzing metabolic differences [6, 7].

Metabolism in GCs is essential for maintaining normal reproductive function. Metabolic changes can affect follicular and oocyte maturation by regulating GC energy metabolism, proliferation, apoptosis, and steroid hormone synthesis [8]. In mice, CCs are enriched in transcripts associated with metabolism and cell proliferation, while MGCs are enriched with transcripts involved in cell signaling and differentiation [9]. However, the metabolites and transcripts of CCs and MGCs in human preovulatory follicles are not well understood.

During follicle development, CCs and MGCs play different roles, and the normal development of these cell lineages is essential for female fertility. The intracellular mechanism of dividing CCs and MGCs under the control of oocytes has not yet been fully resolved [10]. In this study, we aimed to characterize the differences between human CCs and MGCs, especially their metabolomics differences.

## Materials and Methods

### Collection and Isolation of CCs and MGCs

After Ovum pick up (OPU) by transvaginal ultrasound–guided needle aspiration, MGCs were collected from the follicular fluid under a stereomicroscope. CCs were collected after routine denuding of COCs. MGCs float in the follicular fluid are compact, have dark cytoplasm, and are flaky in shape. CCs surrounding the oocyte are loose, have clear cytoplasm, and are cloud-shaped. During the collection process, red blood cells were avoided as much as possible [11]. After collection, MGCs and CCs were washed in a 100-mm cell culture dish with 1X phosphate-buffered saline (PBS), the density gradients technique was used to purify MGCs and CCs using Percoll. The supernatant follicular fluid was first centrifuged and then subjected to 40% and 80% gradient (SAGE) centrifugation. The upper layer contained follicular fluid, the middle ring layer contained MGCs or CCs we wanted to purify, and the bottom layer contained red blood cells [12]. The cells in the middle layer were collected, washed with 1X PBS, and red blood cell lysis buffer (Invitrogen by Thermo Fisher Scientific) was used to lyse the remaining red blood cells. Following washing 3 times with 1X PBS, the cells were cryopreserved in liquid nitrogen until further metabolic analysis [13, 14].

### Cell Culture

Cells were cultured in Dulbecco’s modified Eagle’s medium (DMEM) (Gibco, Thermo Fisher) supplemented with 10% fetal bovine serum and were maintained at 37 °C in 5% CO_2_ and 5% O_2_ humidified air [15]. After 48 h of culture, the culture medium of CCs and MGCs was collected for hormonal detection. The cells for immunofluorescence observations were cultured in confocal dishes until CCs and MGCs formed monolayers.

### Immunohistochemical Technique

Monolayer culture was performed by seeding cell on a glass bottom culture dish with 60% confluence. After that, cells were prepared for immunofluorescence staining according to standard protocols. Cells were fixed in cold 4% paraformaldehyde for 20 min, washed 3 times in 0.5% Triton X-100 in PBS (PBST) for 5 min, and incubated in blocking solution consisting of 1% bovine serum albumin (BSA) in PBST for 2 h. Anti-alpha tubulin (Abbkine, diluted 1:200) and DyLight549 goat anti-mouse (Abbkine, diluted 1:200) antibodies were used as the primary antibody and secondary antibody, respectively. 4′,6-diamidino-2-phenylindole (DAPI) was used for nuclear staining. A laser confocal microscope (NIKON Elipse Ti) was used for observation [16, 17].

### Metabolite Extraction

CCs and MGCs were vortexed for 60 s and sonicated twice for 30 min at 4°C, and then CCs and MGCs were incubated for 1 h at −20 °C for protein precipitation. The collected supernatant was freeze-dried, and the sample was stored at −80 °C before analysis [18].

### Chromatography–Mass Spectrometry Analysis

#### Chromatographic Conditions

The samples were separated using an Agilent 1290 Infinity LC Ultra-High-Performance Liquid Chromatography (UHPLC) HILIC column (25°C; 0.3 mL/min); mobile phase composition A: water + 25 mM ammonium acetate + 25 mM ammonia, B: acetonitrile; gradient elution procedure: 0–0.5 min, 95% B; 0.5–7 min, linear change of B from 95% to 65%; 7–8 min, linear change of B from 65 to 40%; 8–9 min, B maintained at 40%; 9–9.1 min, linear change of B from 40 to 95%; 9.1–12 min, B maintained at 95%; samples were placed in a 4 °C autosampler during the entire analysis [19, 20].

#### Q-TOF Mass Spectrometry Conditions

After the sample was tested, an AB Triple TOF 6600 mass spectrometer was used to collect the first- and second-level spectra. The ESI source conditions were carried out according to the instructions after HILIC chromatographic separation. Ion source gas1 (Gas1) was set to 60, gas2 was also 60, and curtain gas (CUR) was 30. Ion spray voltage floating (ISVF) was ±5500 V (positive and negative modes); the source temperature was 600 °C; the *m*/*z* range of TOF MS scan and product ion scan was 60–1000 Da and 25–1000 Da, respectively; the accumulation time of TOF MS scan and product ion scan was 0.20 s/spectra and 0.05 s/spectra, respectively; the secondary mass spectra were obtained by information-dependent acquisition (IDA); high sensitivity mode was adopted; the declustering potential (DP) was set to ±60 V (positive and negative modes); the collision energy was 35 ± 15 eV; the IDA setting excluded isotopes within 4 Da; and the candidate ions to monitor per cycle was 6 [21–23].

#### qPCR

Total RNA of CCs and MGCs was extracted by a TIANGEN RNA simple Total RNA Kit (TIANGEN), and the RNA concentration was measured by a NanoDrop ND 2000 spectrophotometer (Thermo Scientific). Following cDNA synthesis, a TIANGEN reverse transcription kit (Fastking RT Kit) was used to reverse transcribe the RNA samples. qPCR was carried out by adding ChamQ Universal SYBR qPCR Master Mix (5 μl), forward and reverse primers (0.2 μl, 10 uM), cDNA (1 μl), and then RNase-free water which was added to a total volume of 10 μl. The following three-step PCR amplification protocol was used: 95 °C for 1 min 20 s, 60 °C for 1 min 30 s, and 95 °C for 15 s, for a total of 40 cycles. The housekeeping gene GAPDH was used as an internal reference, and the specific primer sequences are shown in Table 1 [17, 24].

**Table 1:** Gene-related information

Protein	gene	gene's ID	size	Primer Sequences (5'-3')	Tm
Phospholipid-transporting ATPase ABCA1	ABCA1	19	2261	F:CAGGCTACTACCTGACCTTGGT	58.7
				R:CTGCTCTGAGAAACACTGTCCTC	57.6
ATP-binding cassette sub-family G member 1	ABCG1	9619	678	F:GAGGGATTTGGGTCTGAACTGC	58.5
				R:TCTCACCAGCCGACTGTTCTGA	60.6
Low-density lipoprotein receptor	LDLR	3949	860	F:GAATCTACTGGTCTGACCTGTCC	56.9
				R:GGTCCAGTAGATGTTGCTGTGG	58.2
Scavenger receptor class B member 1	SRB1	949	552	F:GGTCCAGAACATCAGCAGGATC	58.1
				R:GCCACATTTGCCCAGAAGTTCC	59.7
Sulfotransferase 2B1	SULT2B1	6820	365	F:CACACTCAACCTTCAGCGCCAT	60.7
				R:GTGAAGTGGTTCTTCCAGTCGC	58.7
Steryl-sulfatase	STS	412	583	F:GCTGGCAAAAGTCAACACGGAG	59.7
				R:GTCCGATGTGAAGTAGATGAGGG	57.1
Aromatase	CYP19A1	1588	503	F:GACGCAGGATTTCCACAGAAGAG	57.8
				R:ATGGTGTCAGGAGCTGCGATCA	57.6
Lysophospholipid acyltransferase 5	LPCAT3	10162	487	F:CAGGATACCTGGTCTGCTTCCA	59.0
				R:TGAAGAGCCAGTGGATGGTCTG	59.4
Cytosolic phospholipase A2	PLA2G4A	5321	749	F:GGATTCTCTGGTGTGATGAAGGC	58.1
				R:CCTTTCTCTGGAAAATCAGGGTG	55.7
Phosphatidylgycerophatase and protein-tyrosine phosphatase1	PTPMT1	114971	201	F:CACCATGAACGAGGAGTACGAG	57.6
				R:GTCATGTCTACTGTGCTGAGCC	58.1
Cardiolipin synthase	CRLS1	54675	301	F:GCAGTCCAGTTAATCTTGGTGGC	58.7
				R:CTGATGCAGCTGTGGTGAAAGC	59.6
Mitogen-activated protein kinase 1	MAPK1	5594	360	F:ACACCAACCTCTCGTACATCGG	59.2
				R:TGGCAGTAGGTCTGGTGCTCAA	61.0
Lymphocyte antigen 96	LY96	23643	160	F:CCCTGTATAGAATTGAAAGGATCC	52.4
				R:TGCGCTTTGGAAGATTCATGGTG	58.3

qPCR primers: *F* forward primer, *R* reverse primer

#### Western Blot Examination

Proteins in CCs and MGCs were extracted using a nuclear protein and cytoplasmic protein extraction kit (Servicebio). Protein concentrations were measured by a BCA protein concentration assay kit (Servicebio). Western blot analyses were performed using standard procedures. The antibodies used are listed in Fig. 8. Image analysis and the densities of the target bands were analyzed by an Alpha software processing system [25, 26].

#### Hormonal Detection

Culture medium from CCs and MGCs was collected and centrifuged at 250 g for 10 min, and the supernatant was collected. Progesterone and estradiol levels were measured using electrochemical luminescence immunoassay using Elecsy Progesterone III and Elecsy Estradiol III kits. The values were analyzed by a Roche Cobas 6000 automatic electrochemiluminescence immunoassay analyzer [27].

### Data Analysis

ProteoWizard was used to convert the original LC-MS/MS data into .mzXML format, and the peak alignment, peak area extraction, and retention time correction were analyzed by the XCMS program. Data were preprocessed by Pareto-scaling and then analyzed by multi-dimensional statistical analysis, including unsupervised principal component analysis (PCA), supervised partial least squares discriminant analysis (PLS-DA), and orthogonal partial least squares discriminant analysis (OPLS-DA). One-dimensional statistical analysis included Student’s *t*-test and multiple variation analysis, and volcano maps were drawn by R software. The qPCR and hormonal detection data were analyzed using SPSS version 20.0 (SPSS Inc., Chicago, CA, USA), and the mRNA expression levels of the target genes are expressed as the mean ± standard deviation. Statistical significance was determined by Student’s *t*-test (*p* < 0.05) [28, 29].

## Results

### Morphological Differences Between CCs and MGCs

**Fig. 1:**
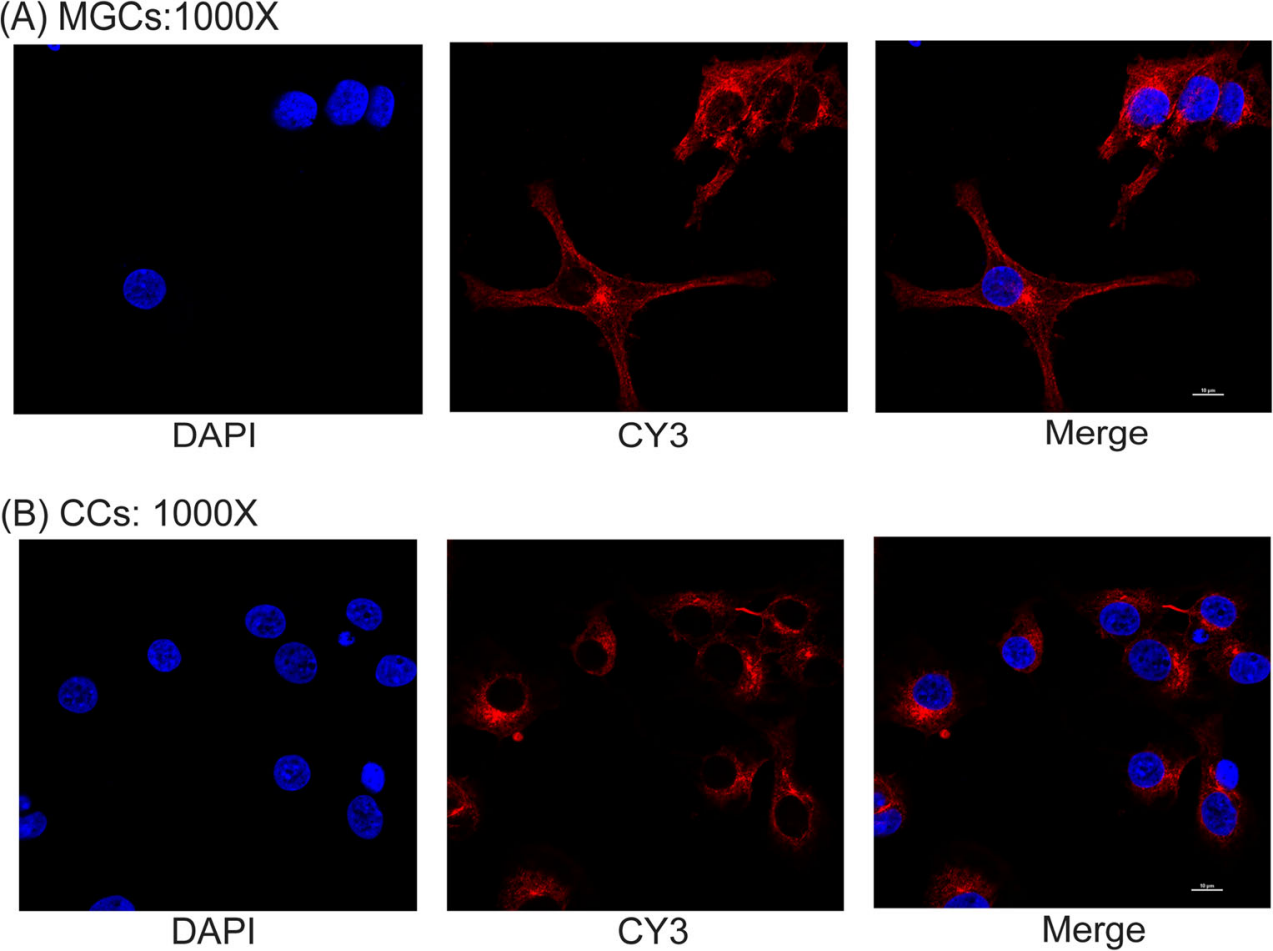
Cultured CCs and MGCs were observed under a laser confocal microscope. A MGCs; B CCs. Magnification: 1000×

Morphological differences between MGCs and CCs were observed under a laser confocal microscope (Fig. 1). MGCs were close to epithelioid cells and fibroblasts and were fusiform, round, stellate, or irregular, while CCs were mostly round in shape.

### Analysis of Spectra/Mass Spectra

**Fig. 2:**
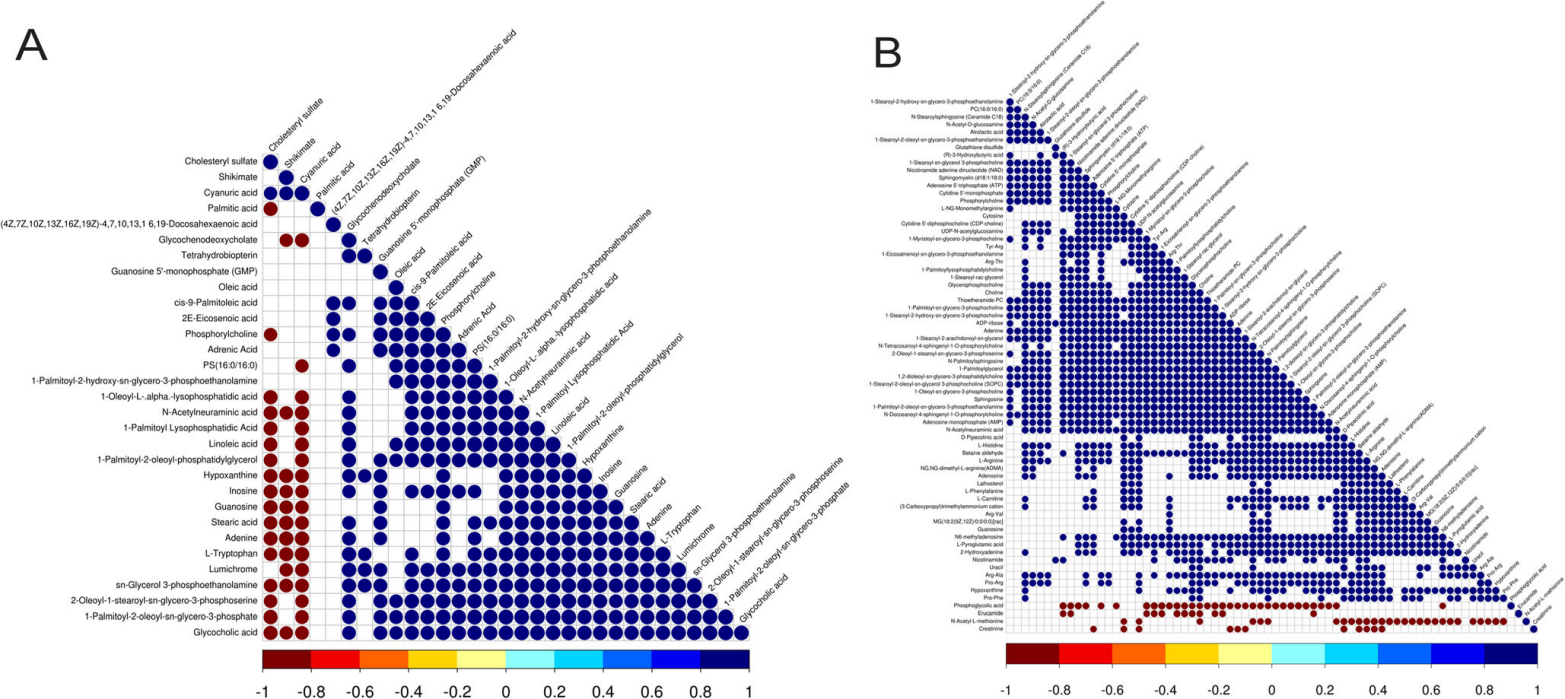
The picture shows the metabolite correlations between CCs and MGCs. **A** is in anion mode, and **B** is in positive mode

**Fig. 3:**
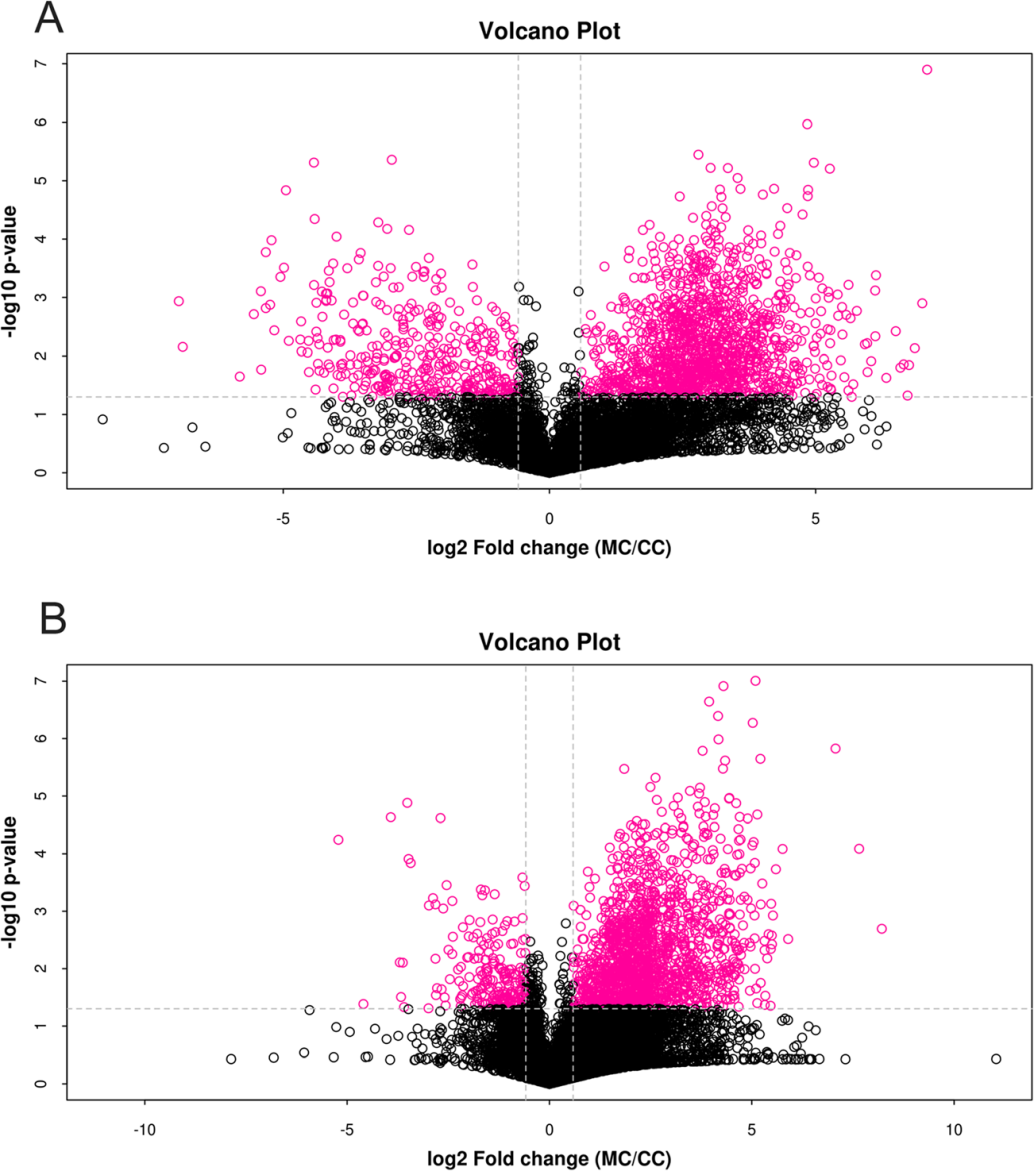
Volcano plots depict differentially detected metabolites and the differences between MGCs and CCs. **A** is in anion mode, and **B** is in positive mode. The x-coordinate in the figure is the logarithm (log 2) of the fold change (MGC/CC), and the y-coordinate is the logarithm (−log 10) of the *P* value significance. The red dots in the figure are metabolites with FC > 1.5 and *P* value < 0.05, that is, the different metabolites screened by univariate statistical analysis

**Fig. 4:**
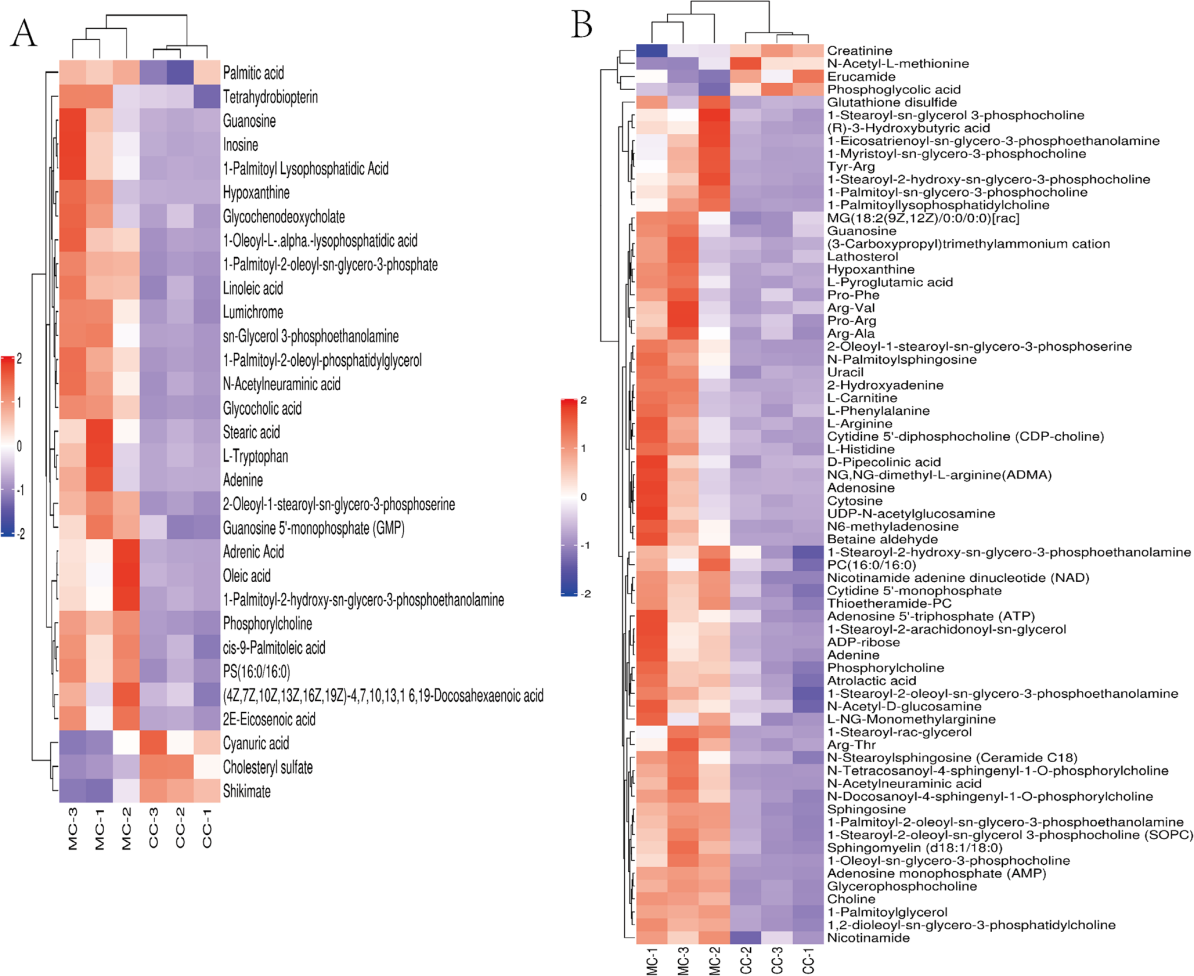
The metabolite contents of MGCs and CCs were analyzed by hierarchical clustering, and the left colored bar indicates the range of fold change values. **A** is in anion mode, and **B** is in positive mode

**Fig. 5:**
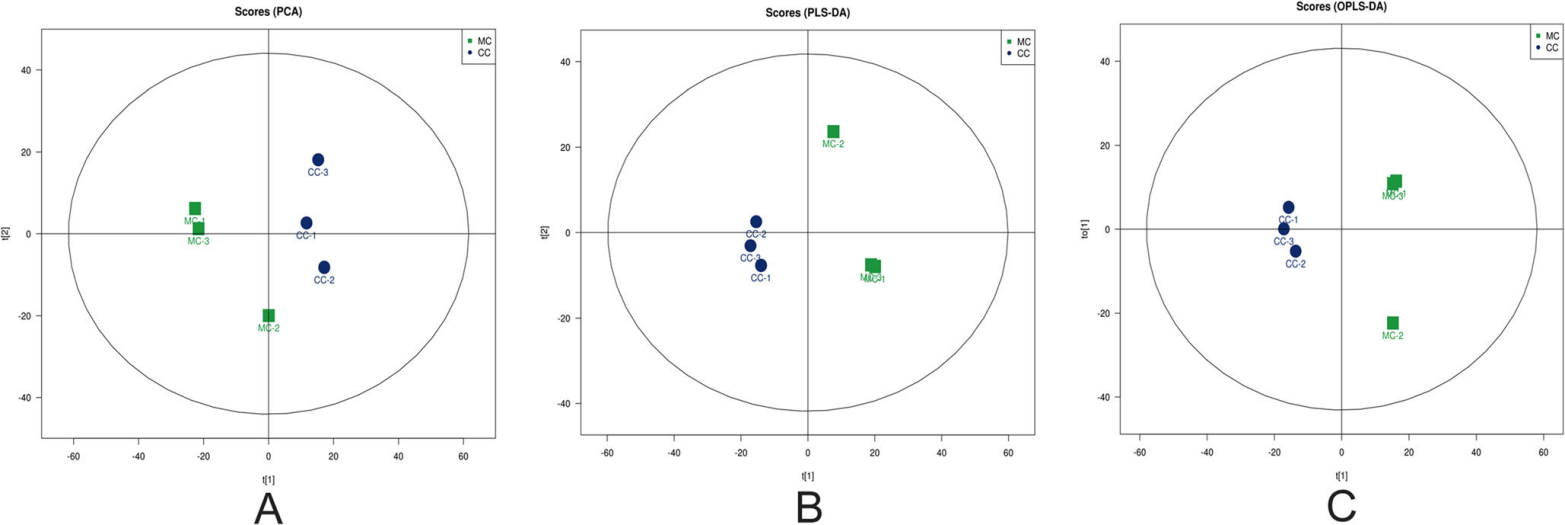
PCA (**A**), PLS-DA (**B**), and OPLS-DA (**C**) plots obtained for metabolite extracts from MGCs and CCs. The x-coordinate represents the first principal component with t [1], and the y-coordinate represents the second principal component with t [2]

There were differences in the metabolic profiles between CCs and MGCs. A total of 101 metabolites were different: 70 different metabolites in positive ion mode and 31 in negative ion mode. There were 94 metabolites upregulated in MGCs: 46 lipids, 18 amino acids, 3 carbohydrates, 7 energy, 15 nucleotides, 3 steroids, and 2 others. A total of 7 metabolites in CCs were higher than MGCs: 4 lipids,1 amino acid, and 2 others (Fig. 2; Fig. 3). The metabolites of variable importance for the projection (VIP) > 1 with multi-dimensional statistical analysis and *P* value < 0.05 with univariate statistical analysis were selected as the significant differential metabolites, while VIP > 1 and *P* < 0.1 were selected as differential metabolites. The metabolites with statistically significant differences in CCs and MGCs were analyzed, resulting in two discrete hierarchical clusters in the heat map (Fig. 4). The PCA model of the overall sample, PLS-DA, and OPLS-DA also showed that there were metabolic differences between CCs and MGCs (Fig. 5).

**Fig. 6:**
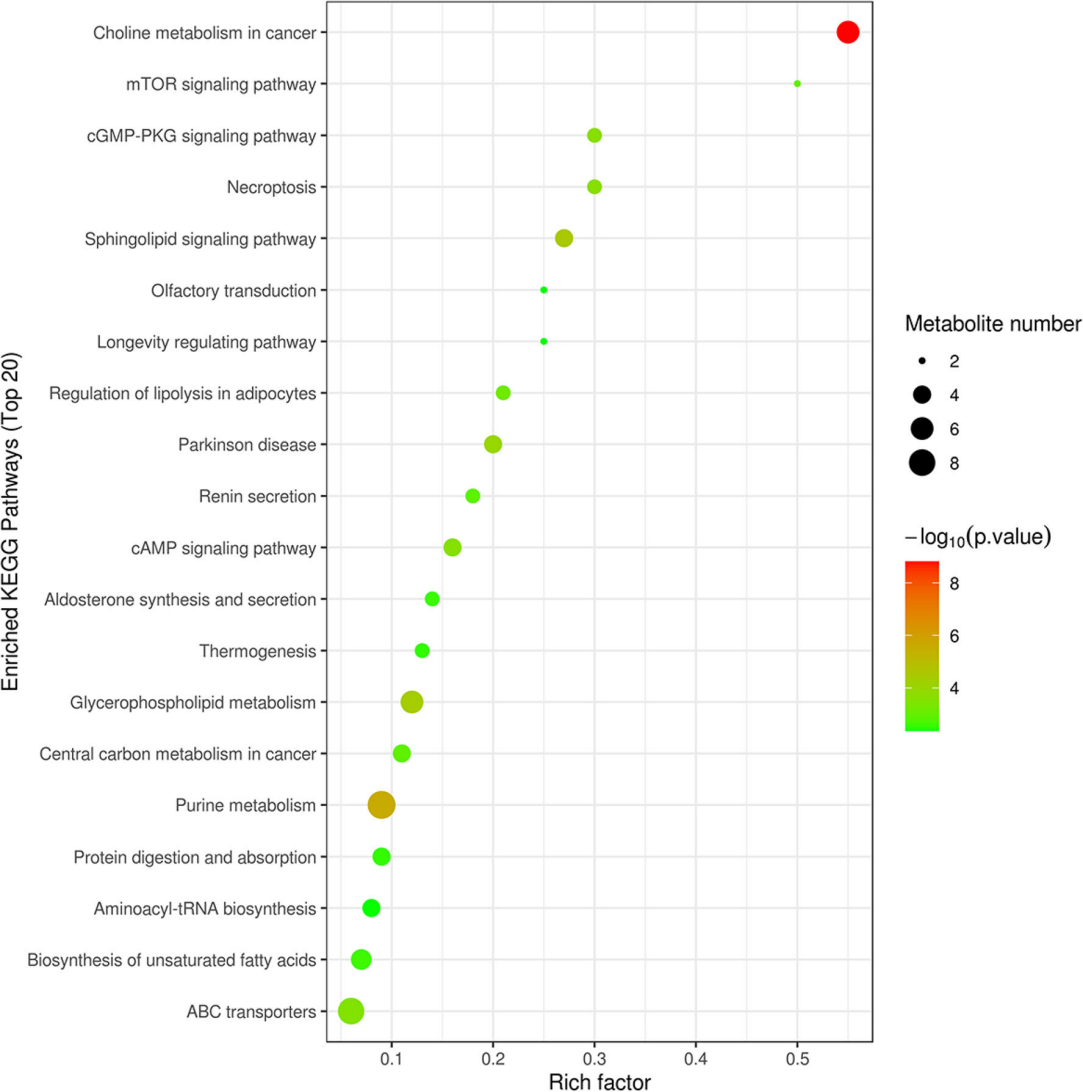
Metabolic pathways in which significant metabolites are involved. The *P* value in the KEGG pathway enrichment results was small (*P* < 0.05), and KEGG pathway enrichment was more statistically significant

MGCs and CCs differentially expressed metabolites by KEGG pathway enrichment analysis. Metabolite hierarchy clustering was significantly different in the sample group. Choline metabolism in cancer, purine metabolism, glycerophospholipid metabolism, and ABC transporters was significantly changed (Fig. 6).

Among the significantly different metabolites upregulated in MGCs, in positive ion mode, Thioetheramide-PC showed the greatest difference between CCs and MGCs, with a fold change (FC) of 4.7. In negative ion mode, the difference in 1-Palmitoyl-2-oleoyl-phosphatidylglycerol (POPG) was most significant, with a FC of 25. Thioetheramide-PC and POPG were highly expressed in MGCs. Of these different metabolites enriched in CCs, erucamide showed the most significant difference between CCs and MGCs in positive ion mode, with a difference value of 1,469,396 and a FC of 0.52, while the largest difference in negative ion mode was cyanuric acid. The difference value between these two was as high as 111,791, and the FC was 0.56. Interestingly, although the content of different metabolites up-regulated in CCs compared to MCGs differed greatly, their FC was not significant. The significantly different metabolites upregulated in CCs were cholesteryl sulfate (CS), phosphoglycolic acid, N-acetyl-L-methionine, and shikimate. Differentially expressed metabolites included cyanuric acid, erucamide, and creatinine.

### Gene Expression in CCs

**Fig. 7:**
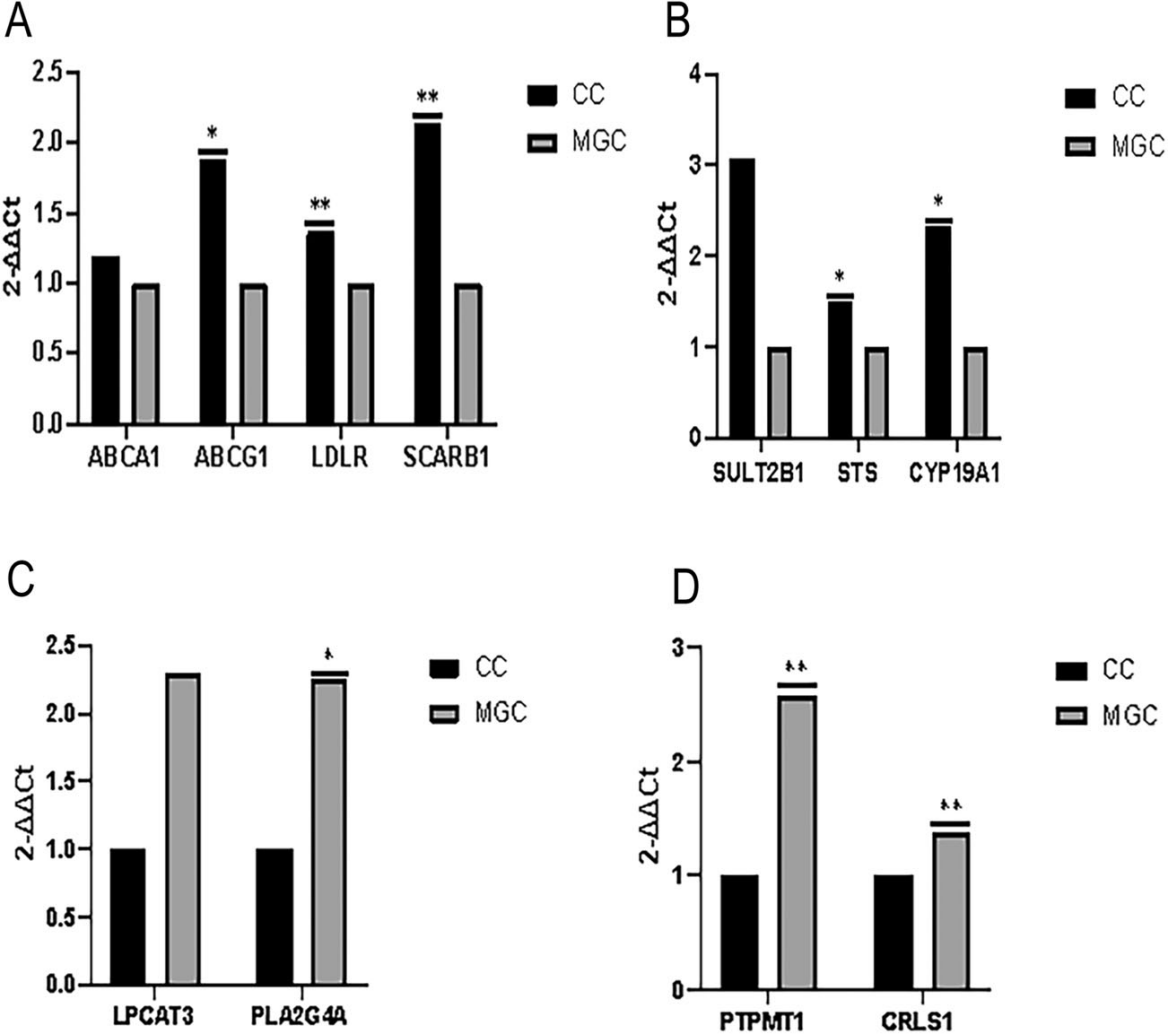
Gene expression of MGCs and CCs was evaluated by qPCR. **A** shows the gene expression of ABCA1, ABCG1, LDLR, and SCARB1; **B** shows the gene expression of SULT2B1, STS, and CYP19A1. In **A** and **B**, the gene expression of MGCs is considered to be 1; **C** shows the gene expression of LPCAT3 and PLA2G4A; **D** shows the gene expression of PTPMT1 and CRLS1. In **C** and **D**, the gene expression of MGCs is considered to be 1. *P < 0.05, **P < 0.01, Student’s *t*-test

By analyzing the LC-MS/MS results, metabolites related to nutrient transport and estrogen synthesis were enriched in CCs, while metabolites related to apoptosis were enriched in MGCs. CS, as the differential metabolite, was enriched in CCs, and the genes involved in CS were selected. The expression of the ABCA1, ABCG1, LDLR, SCARB1, SULT2B1, STS, and CYP19A1 genes in CCs was significantly higher than that in MGCs (*P* < 0.05) (Fig. 7A, B). After 2-ΔΔCt conversion, the expression of ABCA1 in CCs was 1.2-fold higher than that in MGCs. ABCG1 in CCs was higher than in MGCs by 1.88-fold. LDLR was 1.37-fold higher, and SCARB1 was 2.14-fold increased. The expression of SULT2B1 in CCs was 3.08-fold higher than that in MGCs. STS was 1.49-fold higher, while CYP19A1 was 2.33-fold higher.

### Gene Expression in MGCs

From differential metabolites in MGCs, Thioetheramide-PC and POPG were selected as metabolite markers in MGCs. The LPCAT3 and PLA2G4A genes were involved in Thioetheramide-PC. The PTPMT1 and CRLS1 genes were involved in POPG. After 2-ΔΔCt transformation, the expression of genes involved in Thioetheramide-PC and POPG was significantly different (*P* < 0.05). The expression of LPCAT3 in MGCs was 2.30-fold higher than that in CCs, and that of PLA2G4A was 2.25-fold higher. The expression of PTPMT1 in MGCs was 2.59-fold higher than that in CCs, and that of CRLS1 was 1.38-fold higher (Fig. 7C, D).

### Western Blot

**Fig. 8:**
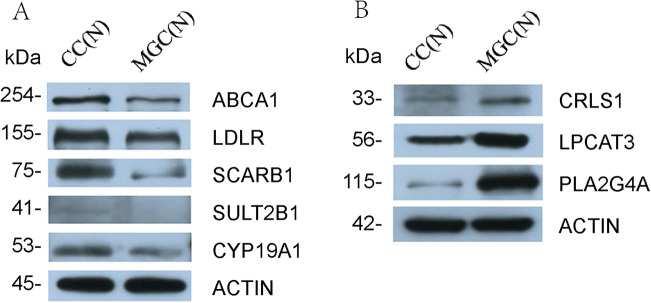
Protein expression in CCs and MGCs. Primary antibodies: mouse anti–human ABCA1 polyclonal antibody (Abcam; diluted 1:3000); rabbit anti–human LDLR polyclonal antibody (PTG; diluted 1:1000); rabbit anti–human SCARB1 polyclonal antibody (Abcam; diluted 1:1000); rabbit anti–human SULT2B1 polyclonal antibody (Abclonal; diluted 1:1000); rabbit anti–human CYP19A1 polyclonal antibody (Abclonal; diluted 1:1000); rabbit anti–human CRLS1 polyclonal antibody (PTG; diluted 1:1000); mouse anti–human LPCAT3 polyclonal antibody (Abcam; diluted 1:1000); rabbit anti–human PLA2G4A polyclonal antibody (Abclonal; diluted 1:1000). Secondary antibodies: goat anti-mouse IgG conjugated with horseradish peroxidase (HRP) diluted 1:3000 in TTBS for ABCA1 and LPCAT3 or goat anti–rabbit IgG conjugated with HRP diluted 1:3000 in TTBS for LDLR, SCARB1, SULT2B1, CYP19A1, CRLS1, and PLA2G4A. The expression of mouse anti–human ACTIN polyclonal antibody (Servicebio; diluted 1:3000) was used as an internal reference. **A** shows the protein expression of ABCA1, LDLR, SCARB1, SULT2B1, and CYP19A1; **B** shows the protein expression of CRLS1, LPCAT3, and PLA2G4A

The protein expression in CCs and MGCs was consistent with the gene detection results. The expression of ABCA1 in CCs was higher than in MGCs by 1.91-fold, LDLR in CCs was higher than in MGCs by 1.40-fold, SCARB1 was higher by 3.65-fold, SULT2B1 was higher by 2.57-fold, and CYP19A1 was higher by 1.23-fold (Fig. 8A). In MGCs, the expression of CRLS1 was 1.79-fold higher than that in CCs, LPCAT3 was 1.78-fold higher, and PLA2G4A was 11.88-fold higher (Fig. 8B).

### Steroid Hormones from CCs and MGCs

**Fig. 9:**
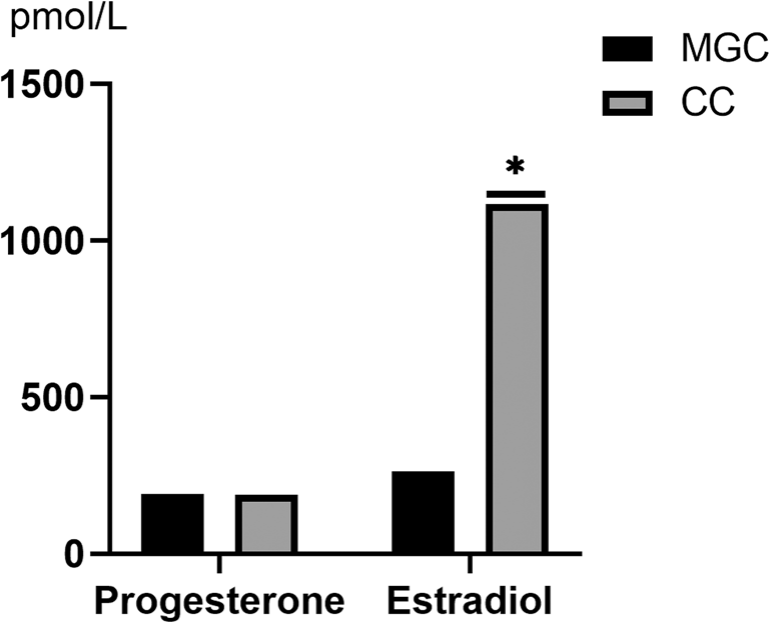
Steroid production in the culture medium from MGCs and CCs. The black color represents MGCs, and the gray color represents CCs. *P < 0.05, Student’s *t*-test

The level of progesterone was not significantly different between CCs and MGCs in the culture medium. Estradiol detection was higher in the culture medium from CCs than that from MGCs (Fig. 9).

## Discussion

Our study presents the first description of the differential metabolomes of two kinds of GCs compartments from stimulated human preovulatory follicles. The analysis of metabolites from CCs and MGCs revealed that differential expression of 101 metabolites with at least a 1.5-fold difference. These results demonstrate that there are differences between CCs and MGCs related to gene expressions, proteins and metabolites in human preovulatory follicles.

Our results showed that the metabolites related to cholesterol transport and estradiol production were enriched in CCs, and the metabolites related to antiapoptosis were enriched in MGCs. Moreover, both CCs and MGCs contained more lipid metabolites, indicating that lipids may an important role in the development of follicles and oocyte growth.

Lipid metabolism in GCs is essential for maintaining normal reproductive function. Lipid metabolism plays an important role in follicular and oocyte maturation by regulating GC energy metabolism, proliferation, apoptosis, and steroid hormone synthesis [8]. In bovine GCs, cell proliferation and ovarian steroid production depend on lipid metabolism, which supports oocyte development and maturation [30]. The involvement of high levels of mobilized oleic acid in follicular fluid in combination with the induced lipid storage in CCs serves to prevent harmful saturated fatty acid (FA) exposure to the oocyte [28, 31].

Interestingly, previous studies have shown that CCs seem to be more important for the growth of oocytes, and MGCs supply more components of the follicular fluid [29, 32, 33]. GCs play a vital regulatory role in initiating follicular growth, atresia, ovulation, and corpus luteum formation. However, the molecular mechanism of GC function is still unclear [34]. Studies have shown that, after GCs differentiate into MGCs and CCs, oocytes acquire the ability to complete meiosis. MGCs can produce natriuretic peptide type C (NPPC), stimulate natriuretic peptide receptor 2 (NPR2) of CCs to produce cyclic guanosine monophosphate (cGMP), promote the diffusion of cGMP diffuses into oocytes, and inhibit cyclic adenosine monophosphate (cAMP)-specific phosphodiesterase (PDE3A) activity and cAMP hydrolysis to maintain prophase blockade of meiotic separation [35]. Although GCs provide necessary nutrition and stimulation for the growth and development of oocytes, oocytes are active regulators of follicular development and not simply passive recipients. There is sufficient evidence that some oocyte secretion factors contribute to the maturation of CCs and MGCs and that growth differentiation factor 9 (GDF9) and bone morphogenetic protein 15 (BMP15) play an important role in the proliferation, differentiation, apoptosis, metabolism, and steroid production of GCs. Studies have shown that BMP15 and GDF9 can promote the biosynthesis of cholesterol in CCs. Recent evidence points to the existence of oocyte-granulosa cell regulatory loops, which drive the development and function of oocytes and follicular somatic cell compartments through complementary signals and metabolic pathways [36, 37].

Usually, people regard MGCs and CCs as the same cells. However, though both CCs and MGCs seem to alike at the initial stage, they play different roles during follicle development [10]. It has been reported that there were significant functional differences in both CCs involved in steroidogenesis and MGCs involved in angiogenesis compartments in human follicles [38]. Differences in animal MGC and CC transcription profiles have also been explored. In mice, differential genomic expression in CCs and MGCs showed that CCs were enriched in transcripts associated with metabolism and cell proliferation, while MGCs were enriched in transcripts involved in cell signaling and differentiation, and >48% of transcripts were higher in CCs than MGCs [9]. However, our results showed that most of the differential metabolites were higher in MGCs than in CCs.

Embryo metabolism is a determinant of viability, nutrients, and metabolites in the culture environment [39]. We are the first to explore the metabonomic differences between human MGCs and CCs. We found that some of the differential metabolites are significant in explaining the mechanism of oocyte maturation.

From our results, metabolites related to apoptosis were enriched in MGCs. This may be due to more susceptibility of follicular fluid for peripheral toxic factors. The follicle formation process is divided into three groups: healthy follicle, migrating follicle, and atresia follicle. Healthy follicles eventually develop into dominant follicles, while follicular atresia is mainly caused by the apoptosis of GCs [40]. GCs are the initial cells that undergo apoptosis in atretic follicles, which precede oocytes and follicular membrane cells, suggesting that they are the initiating factors of follicular atresia [41]. Under normal circumstances, follicular fluid is more susceptible to accumulation of toxic and harmful factors than oocytes. Therefore, MGCs, which are responsible for protecting follicular fluid, are more prone to apoptosis than CCs that protect oocytes. In 1997, Nakahara et al. [42] found that the incidence of apoptotic bodies in MGCs was significantly higher than that in CCs. The apoptosis of MGCs is directly related to female fertility and pregnancy outcome. Fan et al. [5] found that a high apoptosis rate of MGCs is related to a high proportion of empty follicles, low oocyte recovery, and poor oocyte and embryo quality. When the ovarian reserve is normal, the apoptosis of MGCs is significantly reduced, while the apoptosis of CCs is not different [5]. Some studies showed that controlled ovarian stimulation programs may affect the apoptosis of CCs. This may affect the apoptosis and clinical outcome between reduced ovarian reserve and normal ovarian reserve. However, in Nakahara et al.’s [42] prospective cohort, the authors did not observe significant differences in GC apoptosis or follicular fluid hormones between different ovarian hyperstimulation protocols. We found a large number of apoptosis-related substances among the metabolites enriched in MGCs, but the mechanism of their production is still unclear.

From the high level of metabolites in MGCs, L-carnitine can avoid mitochondrial-dependent apoptosis, and choline mediates signal transduction [43, 44]. ADP-ribose is related to the repair of DNA damage [45], and uracil is involved in DNA synthesis [46]. Amino acids participate in energy metabolism and the conversion of the three major metabolites of sugar, fat, and amino acids [47]. Lathosterol participates in the production of cholesterol [48]. Oleic acid has anti-inflammatory and antioxidant effects, and is also related to mitochondrial function and hormone effects [49, 50].

Thioetheramide-PC and POPG were highly expressed in MGCs. Both of these metabolites have been found to participate in anti-inflammatory and antiapoptotic metabolic pathways [51, 52]. This means that compared with CCs, MGCs are more effective in protecting oocytes from damage by harmful components in the follicular fluid and destruction of the follicular microenvironment. In fact, the zona pellucida surrounding oocytes and the extracellular matrix and estrogen produced by CCs are natural antiapoptotic barriers, making oocytes less susceptible to the external environment. This may be the reason why CCs do not need to produce large amounts of antiapoptotic substances [42].

From the qPCR and western blotting results, we found that genes and proteins associated with Thioetheramide-PC, LPCAT3, and PLA2G4A were highly expressed in MGCs. Regarding Thioetheramide-PC, a competitive reversible secretory phospholipase A2 (sPLA2) inhibitor, its functions include cell stabilization, energy source, and storage [51]. The phospholipase A2 (PLA2) family includes strong inflammatory mediators. These enzymes recognize and catalyze the hydrolysis of the sn-2 ester bonds of glycerophospholipids, release free fatty acids such as arachidonic acid (AA) and lysophospholipids, and participate in inflammatory reactions. Many studies have reported that the expression of sPLA2 is related to many pathologies characterized by inflammation. Under pathological conditions, sPLA2 can be induced by a variety of cascades and effector molecules, including inflammatory cytokines and free radicals. Thioetheramide-PC, a PLA2 inhibitor, is considered to be useful for the treatment of neuropathic pain [53]. In bovine GCs, PLA2 is mainly involved in the synthesis of lysophosphatidic acid (LPA) [41]. Among the genes related to the Thioetheramide-PC metabolic pathway, PC produces LPC under the action of cPLA2 and LPCAT, which is a reversible process. The PC and LPCAT3 genes participate in the signaling pathways of the tricarboxylic acid cycle and the pyruvate cycle [54]. The PLA2G4A gene is a member of the cytoplasmic phospholipase A2 group IV family. It plays a major role in the inflammatory response and embryo implantation.

POPG is related to the oxidation reaction, it participates in the lipid peroxidation mechanism. It is also an antagonist of TLR4 and TLR2 activation and intracellular signals, thereby playing a role in preventing inflammation, controlling homeostasis, resisting apoptosis, etc. [52]. POPG contains PTPMT1, CRLS1, TLR4, LY9, CD14, and other genes. PGP generates PG under the action of PTPMT, and PG further generates CL under the action of CRLS1. In the results of our study, we found that PTPMT1 and CRLS1 were significantly higher in MGCs. It has been shown that PTPMT1 silencing increases cell death and chemical sensitivity [55]. CRLS1 may be involved in the pathway leading to apoptosis. The reduction in CRLS1 can lead to abnormalities in CL synthesis, resulting in damage to the integrity or low-activity function of the mitochondrial inner membrane [56]. The TLR4, LY96, and CD14 genes contained in POPG are also closely related to the metabolic pathway of apoptosis.

The LC-MS/MS results in our study showed that the functions of upregulated metabolic factors in MGCs were mostly focused on resisting apoptosis and enhancing cell survival. The gene and protein results related to Thioetheramide-PC and POPG supported this conclusion. This suggests that the metabolites enriched in MGCs enable these cells to play an antiapoptotic effect during follicular development and help oocytes resist the invasion of toxic and harmful substances from the outside. Therefore, the antioxidant capacity of Thioetheramide-PC and POPG may indicate their important potential.

In CCs, we focused on CS. As a signaling molecule and a component of the cell membrane, CS stabilizes the cell membrane. It is also an important regulator of lipid metabolism, cell survival, and apoptosis. As a hydrophilic excretion form of cholesterol, its sulfate-like structure makes molecules more soluble in water, which helps in the transport of nutrients out of the cells to supply follicular fluid and oocytes. We suspect that the intracellular and extracellular transport capacity of CS plays an important role in helping CCs accomplish their functions. CS may be combined with corresponding proteins to achieve the transport of certain substances, such as lipids, and provide nutrition and energy [57]. CS has also been found to be involved in the production of estrogen, which seems to prove why CCs secrete more estradiol, and their ability to secrete estradiol is closely related to the quality of oocytes [58].

The development and maturation of oocytes require a series of factors secreted by somatic cells [59]. During follicle development into antral follicles, a large amount of follicle-stimulating hormone receptor (FSHR) is synthesized on CCs to produce more estrogen and promote follicular development. Dominant follicles are more sensitive to follicle-stimulating hormone (FSH) and produce higher levels of estrogen and the regulatory hormone inhibin. Research has shown that mouse oocytes lack cholesterol synthesis and require CCs to provide cholesterol through their biosynthesis pathways [37]. MGCs and oocytes are separated by CCs, and the sinus cavity is filled with follicular fluid. The insufficient expression of certain phenotypes makes it difficult for MGCs to support the development of oocytes, but MGCs can produce steroid hormones and growth factors. In follicular fluid, these substances secreted by MGCs require CCs to be transported into oocytes through some intermediates, and they play a role in the growth and development of follicles and ovulation. The upregulation of metabolites in CCs is mainly related to substance transport and estrogen production. These functions of CS in CCs are crucial for the growth and maturation of oocytes.

From qPCR and western blotting, our results found that the expression of genes and proteins involved in cholesterol transport and steroid synthesis of CS, such as ABCA1, ABCG1, LDLR, SCARB1, SULT2B1, CYP19A1, and STS, was higher in CCs than in MGCs. ABCA1 and ABCG1 belong to the ATP-binding cassette transporter (ABC) protein family. Their function is to coordinate the export of cholesterol from the cells [60, 61]. Cholesterol is transported from outside the cell to the inside of the cell through LDLR [62]. SR-BI is a type I scavenger receptor B. Through this receptor, macrophages take up modified lipoproteins in the cytoplasm to form foam cells [63].

The general function of SULT2B1 is to participate in the activity of sulfur transferase. It can use 3′-phospho-5′-adenylyl sulfate (PAPS) as a sulfonic acid donor to catalyze many hormones, and sulfonation increases the water solubility of most compounds. In the normal epidermis, cholesterol sulfate is produced by cholesterol under the action of SULT2B1b, and STS desulfurizes cholesterol to form cholesterol [64]. The general function of STS is to participate in catalytic activity, converting sulfated steroid precursors into estrogen during pregnancy [65]. CYP19A1 is an enzyme encoding the cytochrome P450 superfamily. It catalyzes the final step of estrogen biosynthesis. The findings related to those genes and proteins involved in steroid production are similar to the result that the expression of CYP19A1 in uncultured CCs was significantly higher than that in uncultured MGCs [66]. Our result in CCs and MGCs for hormonal secretion indicates that the secretion capacity of CCs for estradiol is stronger than that of MGCs. Our results suggest that estradiol in CCs is higher than that in MGCs, which may be caused by the enrichment of the metabolite CS.

It is commonly believed that CCs and MGCs are from the same origin but at different locations in cells. In recent years, some researchers have conducted studies on the transcription profiles of CCs and MGCs in animals. They found that CCs and MGCs differ in the expression of many important genes [67, 68]. We suspect that there may also be differences between CCs and MGCs in other aspects. Interestingly, in this study, we found that a total of 101 metabolites were different between CCs and MGCs; only 7 were enriched in CCs, and the rest were enriched in MGCs. The functional roles of those metabolites in follicles related to oocyte growth, maturation, and subsequent embryonic developmental potential need to be further explored and studied.

## Conclusion

The results indicate that there were differences between CCs and MGCs in terms of metabolites. Metabolites related to cholesterol transport and estradiol production were enriched in CCs, while metabolites related to antiapoptosis were enriched in MGCs. This finding provides new insight into follicular development and oocyte growth as well as oocyte maturation.

## Data Availability

Data and material are available offline.
